# May Polyphenols Have a Role Against Coronavirus Infection? An Overview of *in vitro* Evidence

**DOI:** 10.3389/fmed.2020.00240

**Published:** 2020-05-15

**Authors:** Giuseppe Annunziata, Marco Sanduzzi Zamparelli, Ciro Santoro, Roberto Ciampaglia, Mariano Stornaiuolo, Gian Carlo Tenore, Alessandro Sanduzzi, Ettore Novellino

**Affiliations:** ^1^Department of Pharmacy, University of Naples Federico II, Naples, Italy; ^2^Department of Clinical Medicine and Surgery, University of Naples Federico II, Naples, Italy; ^3^Department of Advanced Biomedical Sciences, Division of Cardiology, University of Naples Federico II, Naples, Italy; ^4^Department of Clinical Medicine and Surgery, Section of Respiratory Disease, University of Naples Federico II, Naples, Italy; ^5^Chair Staff for Health Education and Sustainable Development, UNESCO, Naples, Italy

**Keywords:** SARS-CoV2, COVID-19 pandemic, polyphenols, antiviral, nutraceutical

## Abstract

The coronavirus infection is constantly diffusing worldwide and the incidence of death is dramatically increasing, representing one of the greatest disasters in human history. Nowadays, no effective therapeutic approaches have been licensed, despite the rising interest of the scientific research in this specific field, and the daily growing number of publications, while the need to find novel strategies is urgent. Evidence in the literature reported the antiviral activity of polyphenols, the largest class of bioactive compounds in nature. Interestingly, a limited number of studies investigated the efficacy of polyphenols from different raw materials, directly against coronaviruses. The present manuscript aimed to report this evidence and provide a viewpoint on the possibility to use it as a start point for the development of novel natural approaches against this viral infection, eventually designing further appropriate researches.

## Introduction

The 2019 coronavirus (SARS-CoV2) pandemic, reported for the first time in Wuhan (China), in December 2019 (1), is rapidly growing with marked morbidity and mortality, resulting in a dramatic socio-economic impact. The diagnosis of SARS-CoV2 infection is based on qualitative Real-time reverse transcriptase-polymerase chain reaction (rRT-PCR) analysis on a nasopharyngeal swab. However, the presence of this virus has been also demonstrated in further tissues, including sputum, feces, bronchoalveolar fluids, and blood, with different viral kinetics (1–3).

Nowadays, no pharmacological treatments have been licensed as effective in terms of both viral titer reduction and/or lowering the virus spread. In several countries, subjects tested positive are receiving off-label and compassionate therapies, including chloroquine, hydroxychloroquine, azithromycin, lopinavir-ritonavir, favipiravir, remdesivir, ribavirin, interferon, convalescent plasma, steroids, and anti–IL-6 inhibitors (4–8). However, a number of adverse effects, including QT prolongation, torsade de pointes, hepatitis, acute pancreatitis, neutropenia, and anaphylaxis have been reported, particularly in patients treated with chloroquine/hydroxychloroquine, azithromycin, and lopinavir -ritonavir. The need to find a strategy that is both effective and safe to face this emergency is urgent.

Polyphenols are the largest class of bioactive compounds present in plants, where are produced as secondary metabolites with protective functions against ultraviolet radiations, pathogen aggression, and oxidative stress protection. Structurally, the term polyphenol refers to the presence of one or more phenolic rings with hydroxyl groups. On that bases, polyphenols can be classified into flavonoid (including anthocyanins, flavones, flavanones, flavonols, isoflavones, and flavan-3-ols), phenolic acids, polyphenolic amides, and other polyphenol compounds (including stilbenes or lignans) (9).

Besides the well-known antioxidant and anti-inflammatory activities of polyphenols, evidence highlighted the antiviral potential exerted by this class of bioactive compounds. In particular, a large number of studies demonstrated the efficacy of polyphenols against several pathogens, including Epstein-Barr virus (10, 11), enterovirus 71 (12), herpes simplex virus (HSV) (13, 14), influenza virus (15), and other virus causing respiratory tract-related infections (16–18). In this context, a great interest has been focused on resveratrol (RSV), whose antiviral mechanisms of actions are mainly attributable to its ability to inhibit the viral replication via (i) inhibition of immediate-early virus protein expression (i.e., ICP-4 and−27), (ii) inhibition of the NFκB signaling pathway, and (iii) activation of the AMPK/Sirt1 axis in the host cell (14).

The present mini-review aimed to report the few promising evidence regarding the potential anti-coronavirus activity of polyphenols, which may serve to drive the research toward the development of novel strategies to counteract the SARS-CoV2 pandemic.

## Polyphenols and Coronavirus

Besides the general mechanisms of action described against various viruses, a limited number of studies investigated the effects of polyphenols directly against coronaviruses. These are *in vitro* studies conducted on different experimental models of infection, using microorganisms belonging to the coronavirus family (Table 1).

**Table 1:** *In vitro* studies investigating the effects of polyphenols against coronavirus.

**Compound(s)**	**Experimental model**	**Treatment**	**Main results**	**References**
Forsythoside A from *Forsythia suspense*	CEK cells infected with IBV	Forsythoside A 0.16 mM, 0.32 mM, and 0.64 mM	(i) dose-dependent viral load reduction, (ii) IBV nucleocapsid protein expression reduction and (iii) dose-dependent inhibition of IBV infection	(19)
(-)-catechin gallate and (-)-gallocatechin gallate	Quantum dots-conjugated oligonucleotide system used for the inhibitor screening of SARS-CoV nucleocapsid proteins		Marked anti-SARS-CoV nucleocapsid protein activity. In particular, (i) dose-dependently ability to attenuate the binding activity at concentrations ≥0.005 μg/ml, (ii) more than 40% inhibition activity at 0.05 μg/ml and (iii) IC_50_ at the same concentration	(20)
Resveratrol	Vero E6 cells infected MERS-CoV	Resveratrol 250–7.8125 μM	(i) cell death reduction at concentrations ranging 250–125 μM, (ii) viral RNA replication inhibition at concentrations ranging 250–31.25 μM, (iii) viral titer reduction at concentrations ranging 250–125 μM, (iv) dose-dependent inhibition of nucleocapsid protein expression at concentrations ranging 250–125 μM and (v) inhibition of apoptosis	(21)
Polyphenols from *Broussonetia papyrifera*	Evaluation of the inhibitory activities of polyphenols against MERS- and SARS-CoV proteases	Compounds were individually tested at concentrations ranging from 0 to 200 μM	All the tested compounds had a dose-dependent inhibitory activity on SARS-CoV protease with an IC_50_ ranging from 30.2 to 233.3 μM	(22)
Crude polyphenolic extract *from Sambucus nigra*	Vero cells infected with IBV	Crude polyphenolic extract 0.004 g/ml	(i) viral replication inhibition, (ii) dose-dependent reduction of virus titers by four to six orders of magnitude at 1.0 and 0.1 MOI, respectively, (iii) inhibition of infection process at an early stage and (iv) altered virus structures and membrane vesicles	(23)

*CEK, Chicken embryo kidney; IBV, infectious bronchitis virus; SARS-CoV, SARS-related coronavirus; IC_50_, Half-maximum inhibitory concentration; MERS-CoV, with Middle East Respiratory Syndrome coronavirus; MOI, Multiplicity of infection*.

**Figure 1:**
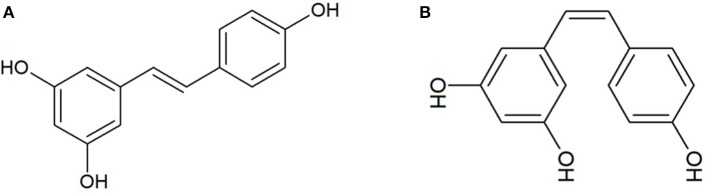
Resveratrol (C_14_H_12_O_3_). **(A)** *Trans*-resveratrol; **(B)** *cis-*resveratrol.

**Figure 2:**
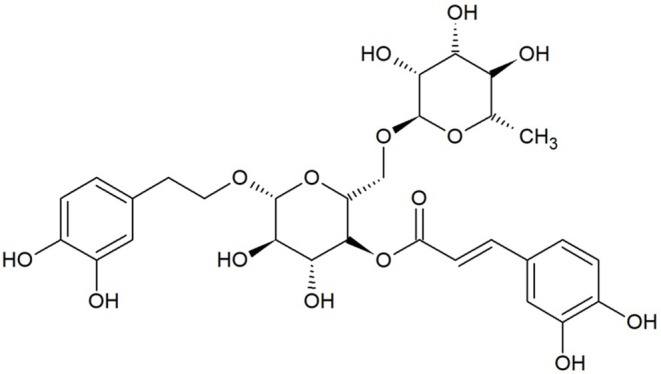
Forsythoside A (C_29_H36O_15_).

In 2017, Lin et al., performed an interesting study aimed to evaluate the anti-coronavirus activity of RSV (3,5,4′-trihydroxystilbene), a 14-carbon skeleton stilbene widely presents in plants, including *Vitis vinifera* and *Polygonum cuspidatum*. RSV exhibits three hydroxyl groups in position 3, 5, and 4′ joined to the two aromatic rings by a double styrene bond that determines the existence of *cis-* and *trans*-RSV isomers (Figure 1) (14). The antiviral activity of RSV was evaluated on Vero E6 cells infected with Middle East Respiratory Syndrome coronavirus (MERS-CoV*)* and treated with RSV at concentrations ranging from 250 to 7.8125 μM. It was demonstrated that RSV (i) reduced the cell death caused by MERS-CoV at concentrations ranging from 250 to 125 μM, (ii) inhibited the viral RNA replication at concentrations ranging from 250 to 31.25 μM, (iii) reduced the viral titer at concentrations ranging from 250 to 125 μM, (iv) inhibited dose-dependently the expression of nucleocapsid proteins at concentrations ranging from 250 to 125 μM, and (v) inhibited the apoptosis, as evidenced by a dose-dependent reduction of the Caspase-3 expression. Thus, this study evidenced the ability of RSV to counteract MERS-CoV infection acting on the main putative mechanisms of action. In particular, according to authors, it was speculated that RSV might be able to (i) activate the ERK1/2 and SIR1 signaling pathways, related to both cell survival and DNA protection, (ii) inhibit the MERS-CoV-induced apoptosis via down-regulation of the FGF-2 signaling pathway, and (iii) reduce the infection interfering with the NFκB-regulated signaling pathway (21). In addition to MERS-CoV, further studies investigated the antiviral potential of polyphenols against infectious bronchitis virus (IBV), another microorganism belonging to the coronavirus family. In particular, the anti-IBV activity of Forsythoside A (FTA) (Figure 2), a phenylethanoid glycoside with chemical formula C_29_H_36_O_15_ isolated from *Forsythia suspense*, was evaluated on chicken embryo kidney (CEK) cells. Cells (both prior to and after virus infection) were treated with FTA 0.16, 0.32, and 0.64mM. It was observed that FTA (i) induced a dose-dependent decrease in viral load, (ii) reduced the gene expression of IBV nucleocapsid proteins, and (iii) dose-dependently inhibited the IBV infection, but had no effect on infected cells (19), suggesting the potential of this bioactive compound as an antiviral agent against IBV. Similarly, the same virus was used to infect Vero cells and the anti-IBV activity of the polyphenols of *Sambucus nigra* was tested. In particular, a crude polyphenolic extract (0.004 g/ml) was used to treat cells 24 h prior to being infected. The pre-treatment with the *S. nigra* polyphenolic extract resulted in the (i) inhibition of the viral replication, (ii) dose-dependent reduction of the virus titers by four to six orders of magnitude at 1.0 and 0.1 multiplicity of infection (MOI), respectively, (iii) inhibition of the infection process at an early stage, and (iv) alteration of both virus structures and membrane vesicles (23). Although the results regarding the anti-IBV potential of *S. nigra* polyphenols are promising, the authors did not characterize the crude extract, thus, the main actors responsible for the antiviral activity cannot be identified. However, previous studies described the phytochemicals contained in *S. nigra* extract, reporting the presence of cyanidin, kaempferol, myricetin, dihydromyricetin, and quercetin derivatives 3-, 4-, and 5-caffeoylquinic acid; kaempferol 3-rutin; rutin; pelargonidin 3-glucoside; isorhamnetin 3-rutin, isorhamnetin 3-glucoside (24–26) and flavonols (5,7,3′,4′-tetra-O-methylquercetin and 5,7-dihydroxy-4-oxo-2-(3,4,5- trihydroxyphenyl)chroman-3-yl-3,4,5-trihydroxycyclohexanecarboxylate) (27). Interestingly, it was also reported that some of these *S. nigra*-derived polyphenols exhibited antivirus activities (27, 28). In this sense, it can be speculated that the antiviral activity is exerted by the phytocomplex including a large number of polyphenolic compounds that, in turn, are eventually responsible for a synergistic effect.

**Figure 3:**
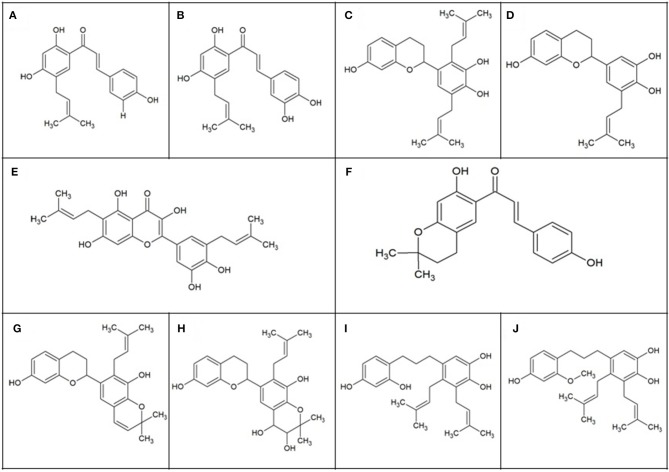
Polyphenols isolated from *Brussonetia papyrifera*. **(A)** Broussochalcone B (C_20_H_20_O_4_); **(B)** broussochalcone A (C_20_H_20_O_5_); **(C)** kazinol A (C_25_H_30_O_4_); **(D)** 3'-(3-methylbut-2-enyl)-3',4,7-trihydroxyflavone (C_20_H_22_O_4_); **(E)** papyriflavonol A (C_25_H_26_O_7_); **(F)** 4-hydroxyisolonchocarpin (C_20_H_20_O_4_); **(G)** kazinol B (C_25_H_28_O_4_); **(H)** broussoflavan A (C_25_H_30_O_6_); **(I)** kazinol F (C_25_H_32_O_4_); **(J)** kazinol J (C_26_H_34_O_4_).

**Figure 4:**
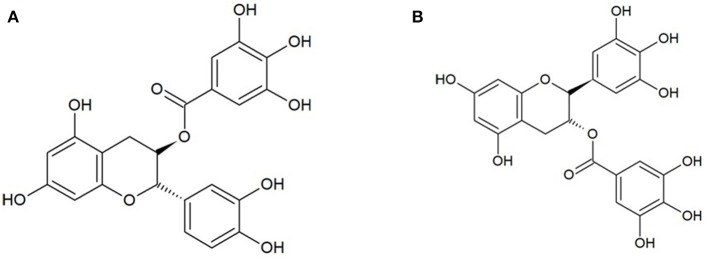
Catechins. **(A)** (-)-catechin gallate (C_22_H_18_O_10_); **(B)** (-)-gallocatechin (C_22_H_18_O_11_).

Besides the investigations on the aforementioned cell lines, two mechanistic studies have been performed to elucidate the specific targets of polyphenols in their anti-coronavirus activity. Particularly, it was tested the anti-MERS- and SARS-CoV activity of ten different polyphenols isolated from *Brussonetia papyrifera*, whose chemical structures are reported in Figure 3. In particular, all the isolated compounds were individually tested at concentrations ranging from 0 to 200 μM, demonstrating their dose-dependent inhibitory activities against MERS/SARS-CoV proteases and finding the half-maximum inhibitory concentration (IC_50_) at concentrations ranging from 30.2 to 233.3 μM (22). On the other hand, a large number of polyphenolic compounds were tested in a quantum dots-conjugated oligonucleotide system for the inhibitor screening of SARS-CoV nucleocapsid proteins. More specifically, the following compounds were studied: quercetin, acacetin, apigenin, baicalein, hesperidin, morin, rutin, naringin, naringenin, (–)-catechin, (–)-catechin gallate, (–)-gallocatechin gallate, diosmin, daidzein, genistein, glycitein, kaempferol, luteolin, myricetin, silibinin, silymarin, orientin, oroxylin A. Among these, (–)-catechin gallate and (–)-gallocatechin gallate (Figure 4) exhibited a marked anti-SARS-CoV nucleocapsid protein activity. In particular, (i) a dose-dependent ability to attenuate the binding activity was observed at concentrations ≥0.005 μg/ml, (ii) at 0.05 μg/ml both the compounds exerted more than 40% inhibition activity, and (iii) at the same concentration was found the IC_50_ (20). These results appear interesting since they clarify specific mechanisms of action and/or putative targets for the antiviral activity of polyphenols. Moreover, they allow to limit the large variety of polyphenolic compounds, leading to identification of such polyphenols for the development of novel natural approaches against coronavirus infection.

Although these studies are appealing and well-conducted, and provide promising results, their careful analysis leads to individuate the following limitations. Firstly, evidence from the anti-coronavirus activity of polyphenols is only provided by *in vitro* studies, although animal-based studies reported the efficacy of polyphenols on other kinds of viruses. No clinical evidence, nor, at least, animal-based studies, are available. However, this lack might be due to the difficulty of designing appropriate studies on animals infected with this kind of virus, due to its dangerousness. Similarly, the need to eventually test natural compounds in humans have not emerged until now, since no many cases were registered. Another limitation is due to the fact that polyphenols are a very large class of bioactive compounds, in which further subclasses can be identified. Although promising, these studies do not provide evidence to establish which subclass of polyphenols deserves to be further investigated. Moreover, taking into account the *in vitro* nature of these studies, no information is provided by authors concerning a possible dose in humans, necessary to design clinical trials.

## Conclusion and Future Perspectives

Overall, this evidence suggests that polyphenols may exert a marked and well-demonstrated activity against coronaviruses, at least *in vitro*, in addition to the previously demonstrated antiviral activity *in vivo*. Studies available in the literature agree in establishing that the reduction of virus titer and the inhibition of nucleocapsid protein expression are their main general mechanisms of action at the base of this promising effect of polyphenols. These elucidated mechanisms are of great interest, since nowadays no effective treatments have been licensed, and the development of novel synthetic drugs against specific coronavirus molecular targets are still far from being achieved.

Despite the aforementioned limitations, these studies should be taken into consideration to design clinical trials. In this sense, the main strength concerning the use of polyphenols in this global emergency is related to the well-established absence of both side effects and drug interactions of such polyphenols with concomitant pharmacological treatments. Indeed, it is well-known that coronavirus-infected subjects are highly prone to develop such respiratory diseases, sometimes complicated by the co-existence of previous cardio-metabolic or chronic diseases. This articulated pathological scenario drastically limits the use of such therapeutic schemes. As an example, the French non-randomized clinical trial showed encouraging results on the efficacy of the combination hydroxychloroquine and azithromycin against COVID19 (6), although both drugs are potentially associated with QT-prolongation (29, 30).

Another relevant point that should be taken into account, is the proper formulation of polyphenol-based nutraceuticals that may be efficient for this scope. Undoubtedly, according to the available studies, potential anti-COVID-19 nutraceutical approaches should contain polyphenols whose effects against coronaviruses have been demonstrated. However, the evaluation of potential synergistic effects between different polyphenols is intriguing. Despite the different structure, indeed, polyphenols share the same chemical features, including the presence of phenolic rings with hydroxyl groups (9). Thus, it could be hypothesized that, although not directly investigated, different classes of polyphenols might exert, at least in part, similar antiviral activities, but eventually with different mechanisms of action. In this sense, according to the studies of Rho (20) and Lin et al. (21), a possible association between RSV and catechins might be speculated for a potential synergy, resulting in hampering the antiviral effect. With this rationale, it should be stressed the importance to investigate the effect of natural polyphenolic extract, rather than the single purified molecules. Interestingly, various plant- or food-derived extracts have been found to be polyphenol-rich matrices for formulation of nutraceutical supplements. Among these, grape pomace extract (GPE) has been reported as an excellent source of bioactive compounds, mainly polyphenols, including RSV, cathechins, and proanthocyanidins (31–33). Notably, evidence indicated the antiviral activity of GPE against various microorganisms, including human immunodeficiency virus type 1 (34), human enteric virus, human norovirus surrogates [feline calicivirus (FCV) F9 and murine norovirus (MNV-19)] (35), hepatitis A virus (36), and hepatitis C virus (HCV) (37). Different mechanisms of actions have been demonstrated, including down-regulation of the HIV-1 entry co-receptor expression (for the activity against HIV) (34), suppression of virus replication via reduction of COX2 expression and regulation of NFκB and MAPK signaling pathways and reduction of virus-induced inflammation (for the anti-HCV activity) (37). Interestingly, two *in vitro* studies investigated the effects of GPE against respiratory syncytial virus, using an airway epithelial A549 cell model (38, 39). In particular, it was demonstrated that GPE interfered with nucleoprotein and fusion protein expression, reducing virus replication. In addition to direct antiviral activity, GPE was reported to be effective in alleviating the pathological complications of the viral infection at respiratory level, reducing the expression of (i) mucins, whose levels increased during the airway mucosa inflammation (38) and (ii) pro-inflammatory interleukins, including IL-1β,−6, and−8 (39). In this sense, the anti-inflammatory potential of polyphenols, mainly exerted via reduction of the interleukin levels, appears noteworthy, and investigating this effect in the context of a virus-induced inflammatory status is intriguing. Overall, this evidence may support the use of polyphenolic extracts, including GPE, for the formulation of potential nutraceutical supplements aimed to counteract the COVID-19 infection. This potential activity might be considered in addition to the well-established antioxidant and anti-inflammatory effects of polyphenols, which may contribute to the general management of respiratory complications of coronavirus infection.

Considering this background, the ideal way to test the antiviral polyphenol effect in humans would be a controlled randomized clinical trial with measurable, reproducible, and clinically relevant outcomes. Most of the current trials are set on the compassionate use of the studied treatment or based on single-arm intervention. Thus, definitive conclusion related to efficacy or safety is hardly deducible. On balance, controlled randomized clinical trials with meaningful clinical outcomes are mandatory to best assess the therapeutic effects and the clinical impact of polyphenol treatment on COVID-19 (40).

## Author Contributions

All the authors contributed to conceptualization, evaluation of the literature and draft-writing.

## Conflict of Interest

The authors declare that the research was conducted in the absence of any commercial or financial relationships that could be construed as a potential conflict of interest.
